# Ten‐year‐old boy with atypical COVID‐19 symptom presentation: A case report

**DOI:** 10.1002/ccr3.3521

**Published:** 2020-11-16

**Authors:** Hamidreza Houshmand, Mahdi Abounoori, Reza Ghaemi, Sara Bayat, Gholamreza Houshmand

**Affiliations:** ^1^ Department of Pediatrics Division of Allergy and Clinical Immunology School of Medicine Urmia University of Medical Sciences Urmia Iran; ^2^ Allergy Research Center Shiraz University of Medical Sciences Shiraz Iran; ^3^ Student Research Committee School of Medicine Mazandaran University of Medical Sciences Sari Iran; ^4^ Department of Internal Medicine School of Medicine Mazandaran University of Medical Sciences Sari Iran; ^5^ Department of Pharmacology Faculty of Medicine Mazandaran University of Medical Sciences Sari Iran

## Abstract

Since reactive arthritis (ReA) and urticaria could be seen in this age group along with atypical COVID‐19 symptom presentation, pediatrics should be familiar with urticarial rashes and ReA in COVID‐19 to enable early diagnoses of infected individuals.

## Graphical abstract

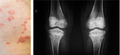

## 1 INTRODUCTION

Herein, we report a case of a ten years old boy with rare clinical manifestations associated with the coronavirus disease 2019 (COVID‐19), in which the first clinical symptoms were urticaria and fever, and then he developed arthritis. The possibility of post‐COVID‐19, reactive arthritis, or postinfectious arthritis can be expected from this virus.

The recent pandemic resulting from a novel coronavirus named severe acute respiratory syndrome coronavirus 2 (SARS‐CoV‐2) has been associated with several different clinical manifestations worldwide. Based on recent studies, fever, dry cough, and fatigue are common symptoms that have been described for this disease.^1^ The coronavirus disease 19 (COVID‐19) maybe mimic the rheumatic diseases. Also, urticarial eruption may manifest in patients with COVID‐19 as the presenting complaint or appear prior to other classical symptoms of COVID‐19.^2^ Herein we reported an unusual presentation of COVID‐19 in a 10‐year‐old boy with arthritis and urticaria.

## 2 CASE REPORT

**Figure 1:**
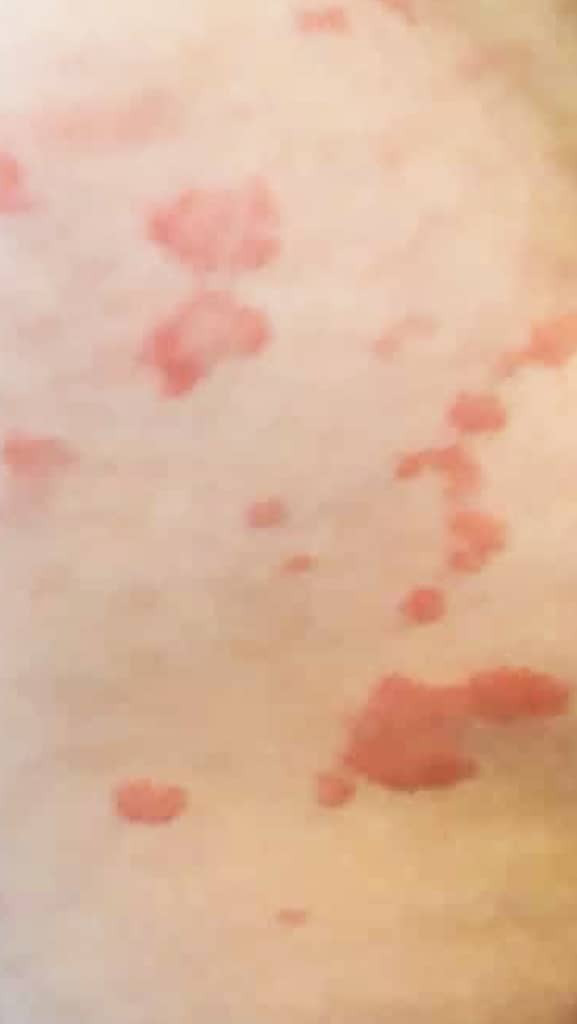
Urticaria view in the abdomen

A 10 years old boy presented with a history of fever and urticaria (Figure 1) from one week prior to admission without a previous history of food or drug allergy. His urticaria was self‐limited, with the recurrence times in a day lasting for one or two hours. Five days before admission, the patient developed pain and swelling in both knees and his right elbow. Then, Cefixime and Acetaminophen were prescribed for him by a pediatrician, but the symptoms remained. His arthritis was associated with morning stiffness, and by movement, his pain becomes attenuated. He had no history of cough, dyspnea, pharyngitis, myalgia, nausea, vomiting, and smelling disorder. In the emergency room, physical examinations (P/E) revealed tenderness, swelling, limitation, and pain of motion in reported sites. The involved joints were warm but without any erythema. Migratory arthritis was not seen in him. The patient's breath sound was normal. The history of COVID‐19 infection did not report in his first‐degree or other family members.

On P/E, body temperature: 37.3°C, blood pressure: 100/60 mm Hg, the pulse rate: 98 beats/min, respiratory rate 17 breaths/min were found without respiratory distress, and O_2_ saturation was 96% on room air. No sign of murmurs or gallops was found on the heart examination. His abdomen was soft and nontender without any organomegaly; the neurologic examination was normal. All cranial nerves were intact.

The mild kidney impairment also was observed with a BUN of 32 mg/dL and creatinine of 1.5 mg/dL and then decreased to 14 and 0.43 mg/dL, respectively, three days after hydration therapy. The sodium and potassium levels were 134 and 3.5 meq/L, respectively. Urine analysis was normal. Stool examinations for ova and parasites and occult blood were normal. Wright's test was negative. Knee joint aspiration was done, which was a dry tap without any joint fluid. Aspartate aminotransferase and alanine aminotransferase were 11 and 17 U/L, respectively, and higher levels of alkaline phosphatase (694 IU/L) were observed.^3^ The Prothrombin Time and INR were intact. Sonographic findings of the abdomen were normal without any organomegaly. Also, it must be noted that the ALP routinely in the children might be elevated due to growth.^4^ The rheumatic factor (RF) and antinuclear antibody (ANA) were normal. Also, D.dimer was less than 200 ng/mL, C3 and C4, Creatine phosphokinase (CPK) test, and Ferritin levels all were in the normal range.

**Figure 2:**
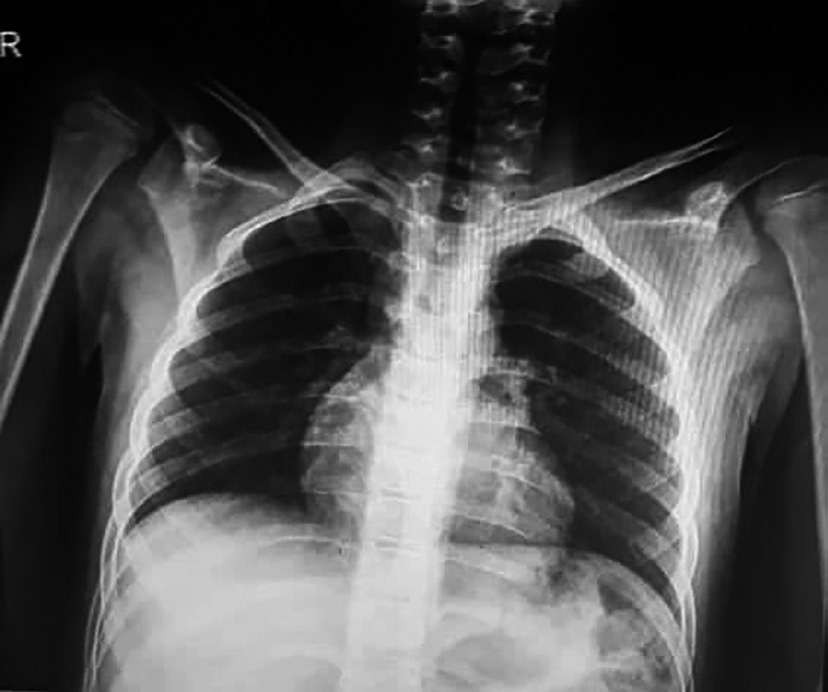
The chest X‐ray presentation

**Figure 3:**
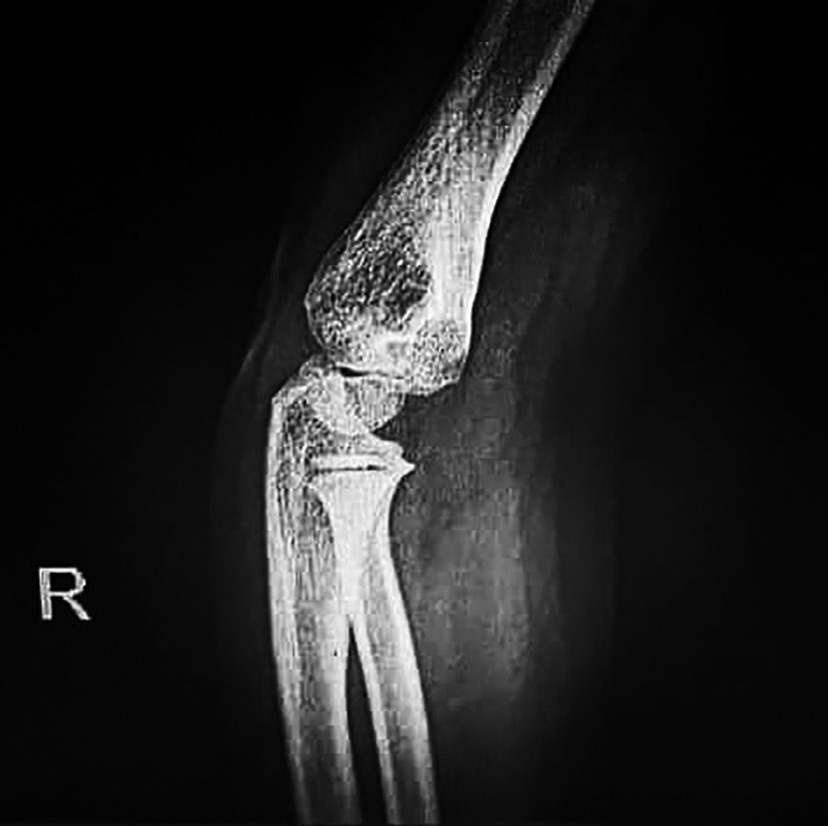
The X‐ray arthritis view in the right elbow is showing the typical bony structures, no visible posterior and anterior fat pad, and no elbow effusion

**Figure 4:**
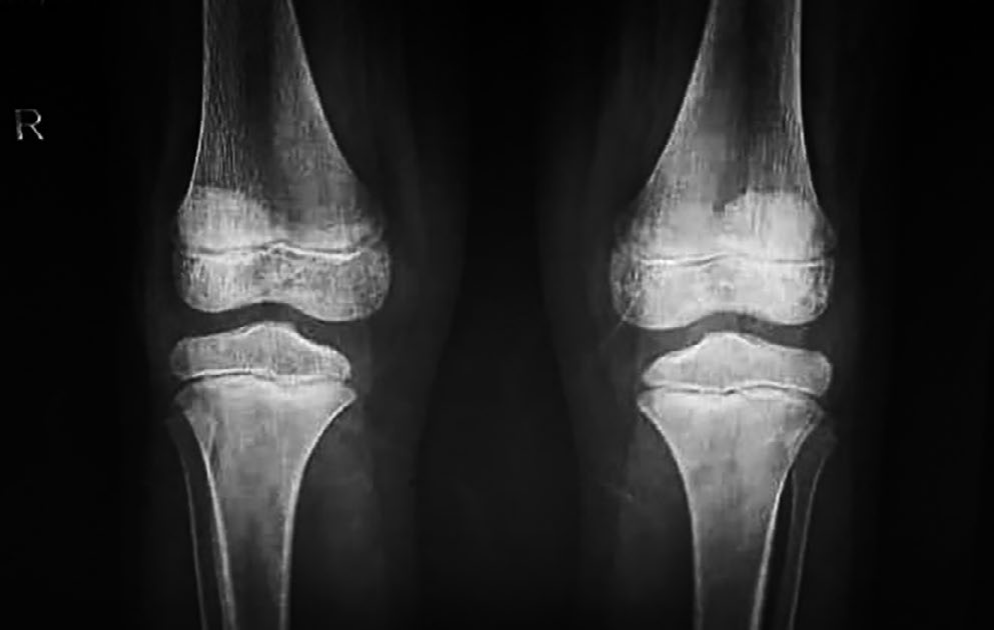
The X‐ray view in both knee joints is showing the bone erosions or spur, joint space narrowing, soft‐tissue swelling, joint effusion, osteopenia, suprapatellar effusion, and synovial thickening

Chest radiographs (Figure 2) did not show the ground‐glass opacity, a typical radiologic manifestation of COVID‐19.^5^ X‐ray graphies of involved joints showed inflammation (Figures 3,4). Although the patient did not declare any travel history or reported known contacts with contagious or infected people, the nasopharyngeal and oropharyngeal swabs tested positive for SARS‐CoV‐2 by real‐time reverse transcriptase‐polymerase chain reaction (rRT‐PCR) assay. So the COVID‐19 has been confirmed for the patient.

The protective COVID‐19 local protocols were performed, with patient hospitalization under isolation and treatment with intravenous Acetaminophen (10 mg/kg PRN), Cetirizine (10 mg per oral (PO) two times a day), Desloratadine (5 mg PO daily), and Hydroxyzine (10 mg PO every night) were used. We did not use any antiviral drugs or Hydroxychloroquine or Interferon Beta.

After 12 days since the initial symptoms and 72 hours after supportive and antihistamine treatments, the patients showed a better general condition, and his kidney condition becomes stable. Arthritis and Urticaria dramatically were removed.

## 3 DISCUSSION

The microorganism's role in acute and chronic arthritis manifestation has not yet been fully elucidated through recent studies. Host aberrant immune response against pathogens and microorganisms colonization in joints could be involved.^6^ Few studies investigated the link between respiratory viruses and rheumatoid arthritis (RA), and particularly in a Korean study, the association between Parainfluenza and Coronavirus with the number of incident RA was confirmed.^7^ Arthritis caused by infectious agents can be due to direct invasion such as septic arthritis or due to a variety of “immune” mechanisms, the term known as postinfectious arthritis. Although many viral or bacterial arthritis results from the direct joint invasion, the term septic arthritis is reserved for those clinical situations in which bacteria are recovered from the synovial fluid. If the fluid is “sterile,” the condition is called postinfectious arthritis or reactive arthritis (ReA) and is defined as sterile arthritis following remote infections.^8^ In this case, the joints aspiration was a dry tap without any joint fluid. After some days, his joint pain and arthritis were responsive to conservative management. As we know, one of the COVID‐19 complications is a cytokine storm. The hyperinflammation in nonimmunodeficient patients due to severe secretion of inflammatory cytokines, macrophage activation syndrome (MAS), or secondary haemophagocytic lymphohistiocytosis (sHLH) could be associated with systemic‐onset juvenile inflammatory arthritis (sJIA) in children.^9^ It must be noted that the dramatic response to both IL‐1 and IL‐6 antagonists were reported in sJIA patients.^10^ Since the patient's condition improved under symptomatic treatment in the absence of biological markers of MAS and HLH, and because the recovery occurred without the use of IL‐1 and IL‐6 antagonists, the possibility of sJIA and sHLH are very low.

Hence reactive arthritis after COVID‐19 infection or associated disorder with this viral infection could be considered. Reactive arthritis before were most reported in sexually transmitted or gastrointestinal infection but less in respiratory bacterial and viral infections. It is important to know that ReA tends to occur most often in men between ages 20 and 50.^11^ Diagnosing the ReA is now based on a diagnostic criterion (Table 1)^12^ that mainly focuses on enteral or urethral infections. In a similar case with the diagnosis of ReA in COVID‐19 positive patient, arthritis occurred precisely three weeks after the infectious episode. Its negative synovial fluid cultures for bacteria strongly led to a clinical ReA diagnosis.^13^ So in a similar condition, our patient also described the relevant arthritis features after the infectious episode, and his synovial fluid culture was negative for bacteria. Also, his joints features for ReA met ReA diagnostic criteria. It must be noted we observed these features in a 10‐year‐old boy, which is a rare age for ReA, and it is rare in the child with COVID‐19.

**Table 1:** Diagnostic criteria for reactive arthritis

Major Criteria	Minor Criteria
(1) Arthritis with 2 of 3 of the following findings	At least one of the following:
‐ Asymmetric	(1) Evidence of triggering infection:
‐ Monoarthritis or oligoarthritis	‐ Positive urine ligase reaction or urethral/cervical swab for Chlamydia trachomatis
‐ Lower limb involvement	‐ Positive stool culture for enteric pathogens associated with reactive arthritis
(2) Preceding symptomatic infection with 1 or 2 of the following findings:	(2) Evidence of persistent synovial infection (positive immunohistology or PCR for Chlamydia)
‐ Enteritis (defined as diarrhea for at least one day and three days to six weeks before the onset of arthritis)	
‐ Urethritis (dysuria or discharge for at least one day, three days to six weeks before the onset of arthritis)	

Note

A “definite” diagnosis of ReA is based on the completion of both major criteria and a relevant minor criterion, while both major criteria characterize a “probable” diagnosis but no relevant minor criterion or one major criterion and one or more of the minor criteria

Cutaneous manifestations are well known to happen in the setting of viral illnesses. The upper respiratory tract infections are the most common trigger of acute urticaria (AU).^14^ In general, no diagnostic workup such as skin testing or immunoassays is necessary for the evaluation of acute urticaria (AU). Further workup may be warranted when allergic causes of AU are suspected.^14^

Urticaria could occur due to vascular disease. The hallmark of Henoch‐schonlein purpura(HSP) is rash and palpable purpura, starting as macules then developing to petechia or larger ecchymosis commonly in the lower extremities. As the classification criteria for the diagnosis of HSP, the patient should have palpable purpura (in the absence of thrombocytopenia) and one or more of the following criteria, including abdominal pain, arthritis or arthralgia, and renal involvement.^4^

Collagen vascular diseases such as Systemic Lupus Erythematosis (SLE) may manifest with urticarial vasculitis. Urticarial vasculitis being considered if urticaria persists at the same location for more than 24 hours. Muckle‐Wells syndrome and Familial Cold Autoinflammatory Syndrome (FCAS) are rare disorders with recurrent urticarial‐like lesions. Muckle‐Wells is characterized by arthritis, progressive nerve deafness, recurrent fever, and elevated ESR. The FCAS is characterized by cold‐induced rash and additional symptoms such as conjunctivitis, sweating, headache.^4^

Our patients had not any urticarial vasculitis ( petechia, purpura, or urticaria which persisted >24 hours), abdominal pain, hematuria, occult blood in the stool, recurrent fever, or other characteristic findings of vasculitis.

Through several experiences in many countries, urticaria skin manifestation has been reported at various ages. Some reported it as a COVID‐19 off‐label medication complication, and some described it in association with the SARS‐CoV‐2 virus and its extrapulmonary problems. Spain physicians reported a 32‐year‐old woman with a pruritic urticarial eruption in the setting of COVID‐19 that manifested several days after starting hydroxychloroquine and azithromycin and which symptomatically responded to antihistamine therapy.^15^ In France, a 27‐year‐old woman described with urticarial eruption along with odynophagia and diffuse arthralgias 48 hours before the onset of fever and chills and COVID‐19 diagnosis.^16^ The onset time of urticaria is also considerable. A literature review reported that 10% of COVID‐19‐associated urticarial rashes present before the classic clinical manifestation. This feature is also observed in our patients who had not most classical features of COVID‐19.^2^, ^17^ Also, in that literature review, all children before 12 years old with COVID‐19 did not have the classical clinical features of COVID‐19, the feature which also described in our case.^17^

Since ReA and urticaria could be seen in this age group along with atypical COVID‐19 symptom presentation, pediatrics should be familiar with urticarial rashes and ReA in COVID‐19 to enable early diagnoses of infected individuals and limit viral spread.

## 4 CONCLUSIONS

Reactive arthritis and postviral infectious urticaria in association with SARS‐COV‐2 infection could be seen in children, and without using any routine COVID‐19 treatments, the disease could be handled. Also, we still do not know that this disease is the causation of reactive arthritis or not, and further studies are needed in this field.

## 5 ETHICAL CONSIDERATION

Written informed consent was obtained from the patient for the publication of this case report as well as accompanying images. A copy of the written consent is available for review by the editor‐in‐chief of this journal.

## CONFLICT OF INTEREST

No conflicts of interest.

## AUTHORS' CONTRIBUTION

HH, RG, and SB: managed the patient. MA, HH, and GH: drafted and revised the manuscript. All authors have approved the final manuscript.

## Data Availability

Data sharing was not applicable to this article as no datasets were generated or analyzed during the current study.
